# Use of iQPR-H_2_O for bone regeneration and its potential in the improvement of osteoporosis

**DOI:** 10.1186/1471-2474-12-227

**Published:** 2011-10-08

**Authors:** Chiming Lee, Meileng Cheong, Wentien Hsiao, Henyu Liu, Chingyu Tsai, Mingfu Wang, Chihhsiung Wu, Kwanghwa Chang, Gowlin Lam, Winping Deng

**Affiliations:** 1 Department of Oral Medicine, Taipei Medical University, Taipei, Taiwan; 2 Department of Radiology, Taipei Medical University Hospital, Taipei, Taiwan; 3 Department of Obstetrics and Gynecology, Cathay General Hospital, Taipei, Taiwan; 4 Graduate Institute of Medical Sciences, College of Medicine, Taipei Medical University, Taipei, Taiwan; 5 Graduate Institute of Biomedical Materials and Engineering Taipei Medical University, Taipei, Taiwan; 6 Department of Food and Nutrition, Providence University, Taichung, Taiwan; 7 Department of Surgery, School of Medicine, Taipei Medical University-Shuang Ho Hospital, Taipei, Taiwan; 8 Department of Physical Medicine and Rehabilitation, Wan Fang Hospital, Taipei Medical University, Taiwan

**Keywords:** osteoporosis, Quantum Persistent Reflection, mouse fibroblasts, senescence-accelerated mice, bone mass density

## Abstract

**Background:**

Current treatments for osteoporosis are associated with various side effects and do not prevent the age-related decrease in osteoblast number. The objective of this study was to evaluate the effects of iQPR-H_2_O on osteogenesis.

**Methods:**

Mouse fibroblast NIH3T3 and pre-osteoblastic MC3T3-E1 cells were cultured in medium prepared with iQPR-H_2_O or unprocessed mineral water (control cells), and proliferation and differentiation were assessed by MTT and alkaline phosphatase assay, respectively. Mineral deposition by the cells was determined using Alizarin red S staining. A mouse model of osteoporosis, ovariectomized SAMP8 mice, was used to evaluate the effects of iQPR-H_2_O on osteogenesis *in vivo*. Mice were given either iQPR-H_2_O or unprocessed mineral water (control group) for four months after which bone mass density (BMD) measurements were made using a bone densitometer and hematoxylin and eosin staining of bone samples.

**Results:**

NIH3T3 cells grown in medium prepared with iQPR-H_2_O exhibited significantly greater proliferation. NIH3T3 and MC3T3-E1 cells demonstrated a significant increase in alkaline phosphatase levels in the iQPR-H_2_O group. MC3T3-E1 cells showed mineralization at day 28. mRNA expression levels of both osteopontin and runt-related transcription factor 2 in MC3T3-E1 cells were higher in the iQPR-H_2_O group compared with the control group. After four months, significantly greater bone regeneration was evident in ovariectomized SAMP8 mice administered iQPR-H_2_O as compared with control group.

**Conclusions:**

iQPR-H_2_O may reduce the symptoms of osteoporosis by improving osteogenesis.

## Background

Osteoporosis is a disease characterized by abnormal bone metabolism caused by an imbalance in bone remodeling, which reduces bone density and increases the incidence of fractures [1,2]. Current treatments for osteoporosis inhibit bone density loss through pharmaceutical and non-pharmaceutical methods [3,4]. Non-pharmaceutical treatments include calcium and vitamin D supplementation [5-8] and regular weight-bearing exercise [9]. Pharmaceutical treatments include bisphosphonates, hormones, parathyroid hormones, and calcitonin, which inhibit resorption or stimulate bone generation [10]. However, adverse effects, including bone necrosis, hyperparathyroidism, vein thrombosis, hot flashes, and foot cramps, have been reported as a consequence of pharmaceutical treatments [11]. In addition, current treatments do not address the fundamental cause of osteoporosis, namely, bone loss due to an age-related reduction in the number of osteoblasts [12].

Infinitesimal Quantum persistent reflection (QPR™) element water(iQPR-H_2_O) is prepared by a steady device of matter energy, which is a patented technology developed by Mr. Lam Gow Lin (New Technology Number M 273565; United States Patent Application 20060099120; http://www.freepatentsonline.com/y2006/0099120.html) based on the theory of controlling "time parameters, quantum movements, and specific spaces" [13]. Specifically, in steady device of matter energy, a reaction column is programmed such that invisible light can cause interference, superposition, reflection, and dispersion for a set amount of time and in a fixed space. Upon treatment, bonds of molecules are broken, thereby producing elements and small molecules after which they are rearranged, increasing the energy state of matter. This treatment can be applied to drinking water, cosmetics, wine, and medical treatments, thereby increasing their energy states.

The present study tested the hypothesis that iQPR-H2O would promote bone deposition in osteoporosis. Therefore, the effects of iQPR-H_2_O on mouse fibroblast (NIH3T3) and osteoblastic (MC3T3-E1) cell lines were examined. NIH3T3 cells have the capacity to differentiate into osteoblasts [12,14,15]. Specifically, cell proliferation, differentiation, and mineral deposition in response to iQPR-H_2_O were assessed. In addition, the effects of iQPR-H_2_O on bone mineral density (BMD) using a mouse model of osteoporosis, ovariectomized SAMP8 (OVX-SAMP8) mice [16,17], was analyzed.

## Methods

### Characterization of iQPR-H_2_O

The water was analyzed by SGS Taiwan Environmental Lab (Taipei, Taiwan). Unprocessed mineral water and iQPR-H_2_O have densities of 0.9961 and 0.9870, respectively. Processing of the water decreased the pH from 6.23 to 6.16. No significant changes in boiling point were observed upon processing. Conductivity, total dissolved solid, salinity, dissolved oxygen, and oxidization-reduction potentials were measured using a YSI Professional Plus handheld multiparameter instrument (YSI Incorporated, Yellow Springs, OH, USA) at a water temperature of 24°C. Although no significant changes in conductivity, total dissolved solid, and salinity were observed, dissolved oxygen were increased from 7.11 to 9.15 mg/L, and oxidization-reduction potentials were reduced from 265.0 to 239.1 after processing. The elements within both types of water were measured by inductively coupled plasma mass spectroscopy (ICP-MS; Table 1).

**Table 1:** Elements in iQPR-H_2_O: a comparison with unprocessed mineral water.

Elements (ppb)	Unprocessed mineral water	IQPR-H2O
Boron	8.87	11.7
Calcium	51.22	21.9
Cobalt	0.22	0.03
Copper	0.23	0.04
Iron	0.38	0.10
Germanium	0.11	0.01
Potassium	37.22	48.50
Magnesium	19.25	23.4
Manganese	0.02	0.06
Sodium	416.5	176
Nickel	0.14	0.02
Phosphorus	6.96	4.38
Selenium	0.33	0.05
Zinc	0.06	0.20
Iodine	36	162
Silicon	173	289
Oxygen	8.50	8.40

### *In vitro *studies

#### Proliferation and viability

NIH3T3 fibroblast (ATCC CRL-1658) and MC3T3-E1 pre-osteoblastic (ATCC CRL-2593) cells (provided by the cell line center, China Medical University, Taichung, Taiwan) were cultured in 96-well plates (2 × 10^3 ^cells/well) in Dulbecco's modified Eagle's medium (DMEM) supplemented with 10% fetal calf serum and prepared with sterilized iQPR-H_2_O or unprocessed mineral water (control medium) for 0, 1, 3, 5, 7, or 9 days. Cell proliferation at the end of each time point was determined using a Cell Growth Determination Kit (Sigma-Aldrich, St. Louis, MO) following manufacturer's instructions.

#### Differentiation

NIH3T3 and MC3T3-E1 cells were cultured in 6-well plates (1 × 10^5 ^cells/well) in iQPR-H_2_O-containing or control media for 0, 1, 3, 5, or 7 days. Differentiation was determined at the end of each time point by assessing alkaline phosphatase (ALP) levels as previously described [18]. Exposure of cells to iQPR-H_2_O was extended to 14 and 28 days after which ALP expression was measured.

#### mRNA Expression of osteopontin and runt-related transcription factor 2

mRNA expression levels of osteopontin (OPN) and runt-related transcription factor 2 (RUNX2) were assessed in MC3T3-E1 cells after 28 days of culture in iQPR-H_2_O-containing or control media. Total RNA was extracted from cells using TRIzol reagent (Invitrogen Life Technologies, Carlsbad, CA, USA). Gene expression levels were measured by reverse-transcription polymerase chain reaction (PCR). The following PCR primers were used:

OPN forward primer, 5'-ATGAGATTGGCAGTGATT-3';

OPN reverse primer, 5'-GTTGACCTCAGAAGATGA-3';

RUNX2 forward primer, 5'-ACTTTCTCCAGGAAGACTGC-3';

RUNX2 reverse primer, 5'-GCTGTTGTTGCTGTTGCTGT-3';

glyceraldehyde 3-phosphate dehydrogenase (GAPDH) forward primer 5', GCTCTCCAGAACATCATCCCTGCC-3';

GAPDH reverse primer, 5'-CGTTGTCATACCAGGAAATGAGCTT-3'.

PCR products were separated by electrophoresis on 1% agarose gels (Agarose I; AMRESCO, Solon, OH, USA) and visualized using ethidium bromide staining.

#### Cell mineralization

MC3T3-E1 cells were cultured in 2-cm diameter plates (1 × 10^4 ^cells/plate) in iQPR-H_2_O-containing or control media for 28 days. Cell mineralization was determined by staining with Alizarin red S (Sigma-Aldrich) as previously described [19].

### *In vivo *studies

#### Animals and preparation

All animal experiments were approved by the Laboratory Animal Research Committee of Taipei Medical University. Female SAMP8 mice (Providence University, Taichung, Taiwan) were maintained in a clean environment with a temperature between 22 to 24°C and 50% humidity and received food (BioLASCO Taiwan Co., Ltd., Taipei, Taiwan) and regular drinking water *ad libitum*. Mice were ovariectomized (OVX) at four months of age to induce osteoporosis following the protocol described by Lo et al. [12]. Two weeks after the surgery (T = 0), OVX-SAMP8 mice received iQPR-H_2_O (experimental group; n = 6) or unprocessed mineral water (control group; n = 6) for four months. BMD was determined each month. After four months, the mice were sacrificed, and bone samples were obtained for further analysis.

### Bone mass density analysis

Baseline BMD was measured two weeks after the SAMP8 mice were ovariectomized (T = 0) and at one-month intervals for four months using the XR-36 bone densitometer (Norland Products Inc., Cranbury, NJ). Data were acquired using scanner software version 2.0.0 (Norland Products Inc., Cranbury, NJ) and analyzed using host software version 2.5.3 (Norland Products Inc., Cranbury, NJ). Changes in spine, knee, and femur BMD (g/cm^2^) were compared with the baseline BMD values.

### Hematoxylin and eosin (H&E) staining

Subsets of six mice were sacrificed two weeks after ovariectomy (T = 0) and one and four months later. After the mice were sacrificed, spine, femur, and tibial bone (knee) samples were removed and fixed in 10% formaldehyde. Samples were softened in decalcification fluid, embedded in paraffin, and sectioned (10 μm thick) for H&E staining. Three sections, which represent three locations in bone, were examined using a microscopic digital camera (Pixera Penguin 150CL Cooled CCD, San Jose, CA), and the area of interest was measured and analyzed.

### Statistical analysis

Normally distributed continuous variables between the control and iQPR-H_2_O groups were compared using independent two-sample t-test. Data are presented as means ± standard deviation (SD). All statistical assessments were two-sided and evaluated at the 0.05 level of significant difference. Statistical analyses were performed using SPSS 15.0 statistics software (SPSS Inc, Chicago, IL).

## Results

### Effect of iQPR-H_2_O on NIH3T3 cell proliferation

**Figure 1:**
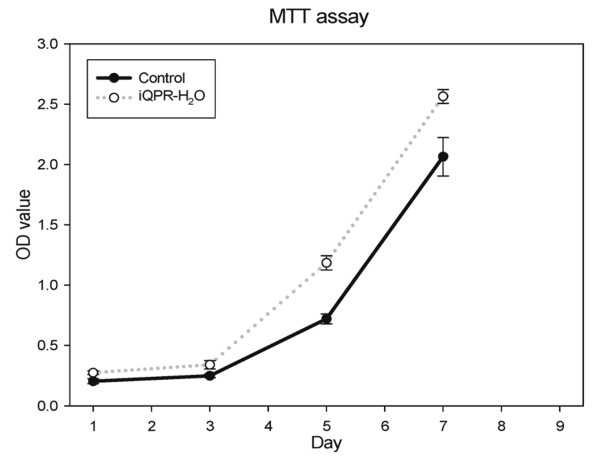
**Effects of iQPR-H_2_O on NIH3T3 cell proliferation**. Cells cultured in medium prepared with iQPR-H_2_O or unprocessed mineral water (control) were analyzed for cell proliferation by MTT assay after 1, 3, 5, 7, and 9 days of culture. Data represent means ± SD of triplicates.

Increased NIH3T3 cell proliferation was observed in cells cultured in medium prepared with iQPR-H_2_O (Figure 1). Cells cultured in media prepared with iQPR-H_2_O attained confluence after seven days whereas control cells were not confluent at this time.

### Effect of iQPR-H_2_O on NIH3T3 and MC3T3-E1 cell differentiation

**Figure 2:**
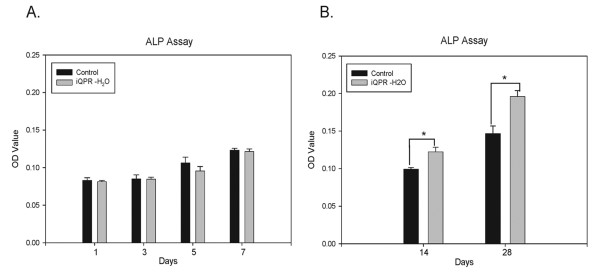
**Effects of iQPR-H_2_O on NIH3T3 cell differentiation**. ALP levels were determined in cells cultured in medium prepared with iQPR-H_2_O or unprocessed mineral water (control) after 1, 3, 5, and 7 days (A), or after 14 and 28 days (B). Data represent means ± SD of triplicates. *P <0.05, as determined by independent two-sample t-tests.

**Figure 3:**
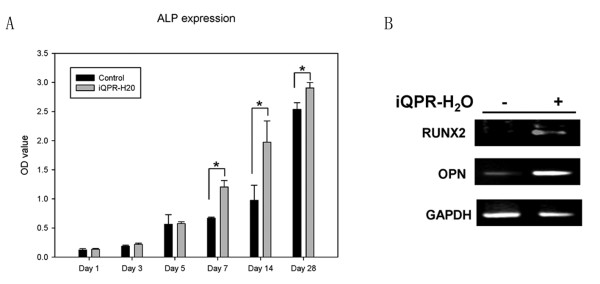
**Effects of iQPR-H_2_O on MC3T3-E1 cell differentiation**. ALP levels were determined in cells cultured in medium prepared with iQPR-H_2_O or unprocessed mineral water (control) after 1, 3, 5, and 7 days, 14 and 28 days (A). Data represent means ± SD of triplicates. *P <0.05, as determined by independent two-sample t-tests. (B) Effect of iQPR-H_2_O on OPN and RUNX2 mRNA expression in MC3T3-E1 cell cultured in the presence (+) or absence (-) of iQPR-H_2_O for 10 days. Expression of GAPDH was determined as a loading control.

The effect of iQPR-H_2_O on NIH3T3 and MC3T3-E1 cell differentiation was determined by measuring ALP levels after short-term (1, 3, 5, and 7 days) or long-term (14 and 28 days) exposure to iQPR-H_2_O-containing medium. There were no significant differences in ALP levels between the iQPR-H_2_O and control groups following short-term culture of NIH3T3 cells (Figure 2A). However, after 14 and 28 days culture of NIH3T3 cells, significantly greater ALP levels were evident in the iQPR-H_2_O group compared with those in the control group (*P *< 0.05, Figure 2B). For MC3T3-E1 pre-osteoblastic cells, there were significantly greater ALP levels evident in the iQPR-H_2_O group compared with the control group from 7 days onwards (*P *< 0.05, Figure 3A). Both OPN and RUNX2 are the markers of osteoblast differentiation. The mRNA expression levels of both OPN and RUNX2 were higher in the iQPR-H_2_O group compared with the control group (Figure 3B).

### Effect of iQPR-H_2_O on MC3T3-E1 cell mineralization

**Figure 4:**
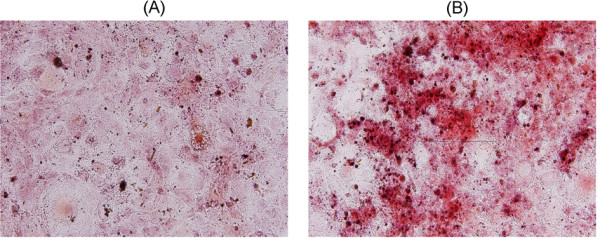
**Effects of iQPR-H_2_O on mineral deposition in MC3T3-E1 cells**. Cells cultured in medium prepared with unprocessed mineral water (control) (A) or iQPR-H_2_O (B) were analyzed for mineralization by assessing Alizarin red S staining (orange-red) using light microscopy after 28 days of culture. 100× magnification.

The effects of iQPR-H_2_O on MC3T3-E1 cell mineralization were assessed using Alizarin red S staining, which identifies calcium-content within tissue. After 28 days, increased Alizarin red S staining was observed in the iQPR-H_2_O group (Figure 4) compared with the control group.

### Effect of iQPR-H_2_O on BMD in OVX-SAMP8 mice

**Figure 5:**
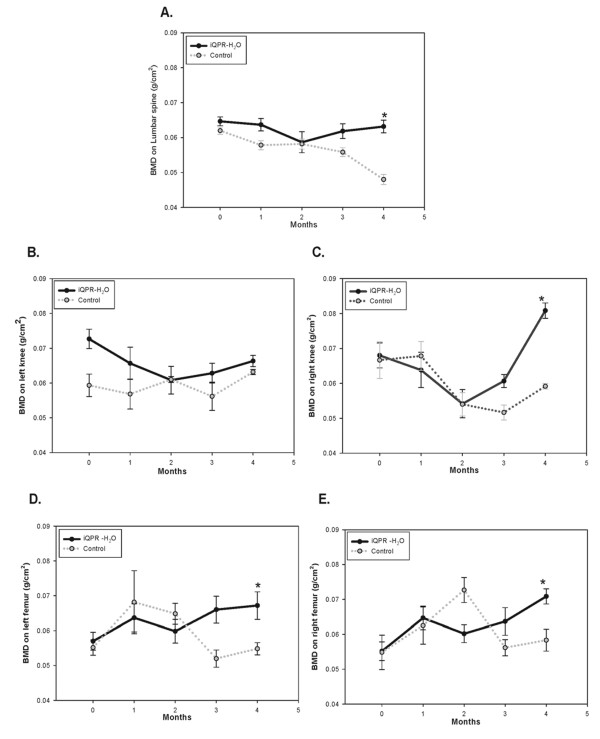
**Effects of iQPR-H_2_O on lumbar spine (A), left knee (B), right knee (C), left femur (D), and right femur (E) BMD in OVX-SAMP8 mice**. Two weeks after ovariectomy, OVX-SAMP8 mice were administered iQPR-H_2_O or unprocessed mineral water (control) at which time baseline (T = 0) BMD values were determined. Thereafter, measurements were made every month for four months. Data represent means ± SD of six mice.*P < 0.05, as determined by independent two-sample t-tests.

Spine, left knee, right knee, left femur, and right femur bone densities were determined in OVX-SAMP8 mice given iQPR-H_2_O or unprocessed mineral water (control). At the zero-, two-, and three-month time points, there were no significant between group differences in bone density; however, after four months, significantly greater bone densities were detected in iQPR-H_2_O-treated mice (Figure 5). Specifically, after four months, BMD levels were significantly higher in spine, right knee, and left and right femur bone samples obtained from mice receiving iQPR-H_2_O as compared with those obtained from control mice (all *P *< 0.05; Figure 5A, C, D, E). No difference in left knee BMD was observed between the groups (Figure 5B).

### Effect of iQPR-H_2_O on bone density in OVX-SAMP8 mice as determined by H&E analysis

**Figure 6:**
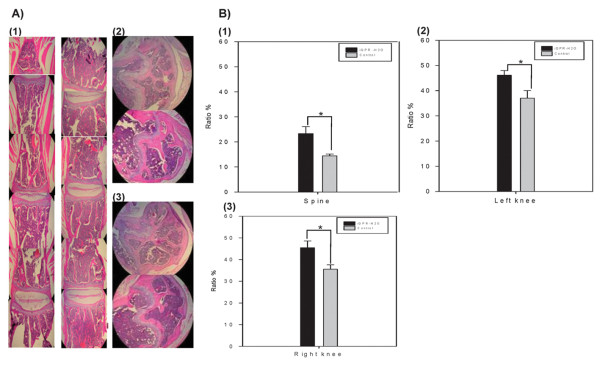
**Effect of iQPR-H_2_O on bone density as determined by H&E staining**. Bone samples were obtained from OVX-SAMP8 mice given iQPR-H_2_O or unprocessed mineral water (control) for four months and stained with H&E. (A) Representative images of (1) lumbar spine (left and right panels), (2) left knee (top and bottom panels), and (3) right knee sections (top and bottom panels) from experimental and control mice, respectively. (B) Bone density as quantified using Pixera Penguin 150CL Cooled CCD and software. (1) lumbar spine, (2) left knee, and (3) right knee. Data represent means ± SD of six mice.* *P *< 0.05, as determined by independent two-sample t-tests.

Bone samples were obtained from OVX-SAMP8 mice given iQPR-H_2_O or control water for four months and stained with H&E. As shown in Figure 6A, representative microscopic images of (1) lumbar spine (left and right panels), (2) left knee (top and bottom panels), and (3) right knee sections (top and bottom panels) were analyzed from experimental and control mice, respectively. After four months, mice receiving iQPR-H_2_O exhibited significantly higher BMDs in the spine and both knees compared with control group mice (all *P *< 0.05; Figure 6B). In addition, bone tissue biopsies at five locations revealed increased trabecular bone in iQPR-H_2_O-treated mice (data not shown).

## Discussion

The effects of iQPR-H_2_O on cell proliferation, differentiation and mineral deposition as well as bone formation were analyzed in this study under *in vitro *condition using NIH3T3 fibroblast and MC3T3-E1 osteoblastic cell lines, and under *in vivo *condition using mouse model of osteoporosis as described previously [12]. NIH3T3 cells treated with iQPR-H_2_O displayed an increased ability to proliferate. Both NIH3T3 and MC3T3-E1 cells in iQPR-H_2_O-containing medium showed higher levels of differentiation compared with control cells. *In vivo *studies revealed that iQPR-H_2_O increased BMD in OVX-SAMP8 mice, indicating that under long-term induction, osteoblasts may be capable of differentiation and improving the symptoms of osteoporosis.

MC3T3-E1 cells showed a sign of differentiation i.e. significantly higher ALP level than control, 7 days after culture in iQPR-H_2_O-containing medium. Further, expression of both OPN and RUNX2 mRNA was higher in the iQPR-H_2_O group compared with the control group after 28 days of culture. This observation is similar to that from a previous report showing that ALP levels were significantly higher 7 days after MC3T3-E1 cells were stimulated with ascorbic acid and b-glycerophosphate [20]. OPN has been reported to modulate both mineralization and bone resorption, and RUNX2 is one of the major regulators of OPN in osteoblastic cells [21]. Increased expression of both OPN and RUNX2 28 days after the culture suggests that iQPR-H_2_O may have promoted differentiation and mineralization of MC3T3-E1 cells. In contrast to MC3T3-E1 cells, increased differentiation of NIH3T3 cells was only observed in cells cultured for 14 to 28 days in the presence of iQPR-H_2_O. There were no statistically significant differences in ALP expression between the iQPR-H_2_O-treated and control cells at earlier time points (ie, within 7 days). This may be explained by the cells being in a proliferative state, which has been reported to delay differentiation [22], within the first 7 days of culture.

Previous studies of bone remodeling have reported that there are five stages to this process: activation, resorption, reversal, formation, and quiescence. Progression from activation to resorption was found to take place over two weeks, whereas three to four months transpired between reversal and formation, and finally quiescence [23,24]. In the present study, OVX-SAMP8 mice exhibited increased bone density after four months treatment with iQPR-H_2_O. This time frame is comparable to that over which bone remodeling takes place. H&E findings corroborated the BMD analysis findings.

Bone densities in iQPR-H_2_O-treated OVX-SAMP8 mice declined between zero and two months and then rebounded during the third month. This presumably reflects osteoclast resorption of old bone caused by secretion of ALP, collagenase, lysosomal enzymes, and proteases, followed by stimulation and recruitment of osteoblasts into the site of osteoporosis. The formation period is a slow phase, which takes approximately 35 to 40 days, which coincides with the BMD changes observed over the four-month period in the present study.

Of note, we found that left knee BMD, as determined by bone densitometry was not significantly increased in mice treated with iQPR-H_2_O compared with control. This is a very puzzling finding for which we do not have an explanation. Indeed, the lack of a significant change in the left knee BMD as determined by bone densitometry is inconsistent with the findings for all other measures of BMD and bone density. We can only assume that the observed BMD findings are a statistical anomaly.

iQPR-H_2_O has approximately 60% less calcium than unprocessed mineral water. It is well known that inadequate intake of vitamin D and calcium can lead to reduced calcium absorption, a higher rate of bone remodeling, and increased bone loss. Although we have not assessed calcium homeostasis in mice treated with iQPR-H_2_O, we do not believe that a 60% reduction in the calcium content of drinking water would significantly alter calcium homeostasis given that the vast majority of calcium is obtained through the diet.

The mechanism by which iQPR-H_2_O influences bone remodeling remains to be investigated. iQPR-H_2_O may have increased the uptake of bone-associated elements from the diet in the experimental animals. As compared with unprocessed mineral water, iQPR-H_2_O had relatively high concentrations of magnesium, zinc, and silicon. Although the mechanism associating with the change in the level of elements is still unknown, this outcome is reproducible.

The importance of magnesium, zinc, and silicon and their deficiencies on BMD has been demonstrated in numerous studies. For example, magnesium deficiency has been associated with hypertension, cardiac arrhythmias, myocardial infarction, hypokalemia and hypocalcemia [25]. Epidemiological studies revealed that BMD of the spine was significantly correlated with magnesium intake in premenopausal women [26]. A cross-sectional study reported a significant correlation between magnesium intake and hip BMD men aged 69-97 years [27].

Several studies have assessed the effect of magnesium therapy for osteoporosis with varied results. Specifically, a prospective study in which postmenopausal osteoporotic women were given 250 mg of magnesium per day reported a significant 2.8% increase in the bone density of the distal radius at one year; however, no significant differences were shown at two years, which may be due to the small number (n = 10) of patients completing the study [28]. A retrospective study in which postmenopausal women received 200 mg of magnesium per day resulted in a small, non significant 1.6% rise in lumbar spine bone density, and no differences were reported in the femur [29]. In another small uncontrolled trial, a significant increase in the bone density of the proximal femur and lumbar spine was found in patients with gluten-sensitive enteropathy who received 504-576 mg of magnesium per day for two years [30].

Discrepant effects of zinc on bone progenitor cells have been reported. For example, in bone marrow cells, the highest ALP activity was observed when they were cultured on the ceramics with 1.26 wt% zinc; the ceramics released zinc ions at concentrations ranging from 2.2 to 7.2 μg/mL into the culture medium [31]. Zinc ions were incorporated into mineralized areas produced by bone marrow cells. In addition, bone marrow cells cultured in medium supplemented with 100 μM ZnCl_2 _had significantly increased the ALP activity [31]. However, Popp et al. [32] reported no differences in rat bone marrow stromal cell number, ALP activity, total protein content, collagen synthesis, or matrix mineralization in response to zinc supplementation.

Associations between silicon intake and BMD have also been seen. The most readily bioavailable source of silicon in the diet includes drinking water as well as other fluids, accounting for ≥ 20% of the total dietary intake [33]. Beneficial effects on bone and other connective tissues have been observed upon silicon supplementation. For example, dietary silicon intake is associated with higher bone mineral density (BMD) [34]. In osteoporotic subjects, silicon supplementation with monomethyl trisilanol resulted in increased bone volume and increases in femoral and lumbar spine BMD [29].

One limitation of this study is that increasing the energy state of water after processing with steady device of matter energy was not measured quantitatively, and that the mechanism associating with the change in the level of elements after the process was unknown. A further limitation is that ALP activity levels were found to increase with time in control MC3T3-E1 cells. This finding is puzzling and suggests that the cells were undergoing spontaneous differentiation under the culture conditions without extracellular stimulation.

## Conclusion

This is the first study to analyze the effect of iQPR-H_2_O on bone remodeling in animals. iQPR-H_2_O ameliorated symptoms of osteoporosis and improved bone matrix. Further studies are needed to analyze the mechanism by which iQPR-H_2_O exerts its effects on osteogenesis and to determine if the beneficial effects of iQPR-H_2_O on osteogenesis are mediated through increased intake of magnesium, zinc, or silicon. The effects of iQPR-H_2_O on endogenous stem/progenitor cells in bone marrow will also be analyzed. iQPR-H_2_O may ultimately prove to be a useful treatment for osteoporosis.

## Competing interests

The authors declare that they have no competing interests.

## Authors' contributions

CL performed research and design; HL performed research and design; WH performed research and design; CT performed research and design; EL performed research and design; CL planned and directed the research; MW planned and directed the animal research; GL planned and developed the QPR technology; CL guarantor of integrity of the WD guarantor of integrity of the entire study and wrote the manuscript. All authors read and approved the final manuscript.

## Pre-publication history

The pre-publication history for this paper can be accessed here:

http://www.biomedcentral.com/1471-2474/12/227/prepub
